# Adenocarcinomas of the esophagogastric junction: experiences at a single institution in China

**DOI:** 10.1186/1477-7819-11-155

**Published:** 2013-07-13

**Authors:** Hao Zhang, Wei Wang, Yao Cheng, Yongchun Song, Kun Zhu, Chengxue Dang

**Affiliations:** 1 The Department of Surgical Oncology, College of Medicine, The First Affiliated Hospital, Xi’an Jiaotong University, 277 W. Yanta Road, Xi’an, Shaanxi, 710061 China

**Keywords:** Adenocarcinoma of the esophagogastric junction, Resection, Prognosis

## Abstract

**Background:**

The incidence of adenocarcinoma of the esophagogastric junction is increasing. This study aims to evaluate the clinicopathological features of Chinese patients with adenocarcinoma of the esophagogastric junction and to define prognostic factors.

**Methods:**

We retrospectively reviewed a database of 382 consecutive patients with adenocarcinoma of the esophagogastric junction at the First Affiliated Hospital of Xi’an Jiaotong University from January 2005 to March 2010. All patients were classified according to the Siewert’s classification and staged according to the latest edition of the American Joint Committee on Cancer categories.

**Results:**

Six of the 382 patients had type I adenocarcinoma, 220 had type II, and 156 had type III. There was no significant difference in the overall survival of different Siewert subtypes. According to the multivariate analysis, pathological lymph node stage, age, and the existence of lymphovascular invasion were the independent factors of prognosis of adenocarcinoma of the esophagogastric junction following surgery.

**Conclusions:**

On the data, the distribution of the three types of tumor was found to be different from that reported in Western countries. Lymph node metastasis, lymphovascular invasion, and age were significant and independent factors for poor prognosis after R0 resection for adenocarcinoma of the esophagogastric junction.

## Background

In Western countries, there has been a dramatic increase in the incidence of adenocarcinomas of the esophagogastric junction (AEG). On the other hand, Eastern countries have not experienced such an increase [1-4]. The current International Union against Cancer (UICC) TNM classification of malignant tumors is the first to define the classification of adenocarcinomas of the esophagogastric junction. According to the UICC, a tumor with an epicenter within 5 cm of the esophagogastric junction (EGJ) and extension into the esophagus, is classified and staged according to the esophageal scheme. Tumors with an epicenter greater than 5 cm from the EGJ or those within 5 cm of the EGJ without extension into the esophagus were staged using the gastric carcinoma scheme [5]. In 1996, Siewert and Stein published a classification of AEG which was later approved at the second International Gastric Cancer Congress in Munich in April 1997. According to this classification, a tumor could be identified into three types (type I, type II, and type III) in terms of the anatomic location of the tumor center. Specifically, type I is adenocarcinoma of the distal esophagus with the epicenter located within 1 cm and 5 cm above the anatomic EGJ; type II is true carcinoma of the cardia with the tumor epicenter within 1 cm above and 2 cm below the EGJ; type III is subcardial carcinoma with the tumor epicenter between 2 and 5 cm below the EGJ [6]. Before the UICC classification, type I was usually classified with the esophageal scheme, and types II and III were classified with the gastric carcinoma scheme [4,7,8].

In China, institutes seldom focus on this special anatomic location, they often treat AEG as distal esophagus or proximal gastric cancer. Therefore, this study analyzes the clinicopathological characteristics and factors which could affect overall survival.

## Methods

### Patients

We retrospectively reviewed a database of 382 consecutive patients of distal esophageal adenocarcinoma, adenocarcinoma of the cardia, and proximal gastric adenocarcinomas at the First Affiliated Hospital of Xi’an Jiaotong University from January 2005 to March 2010. The retrospectively collected data of these patients included demographic parameters, histopathologic tumor characteristics, operation methods, and survival time. The median follow-up for the cohort was 26.7 months (range 1–75 months). We measured the distance from the cardiac dentate line to the oral top of the tumor base on the CT scanning, gastroscopy, operative, and pathological findings, and defined it as the length of esophageal invasion [9]. The eligibility criteria were: i) patients without a history of prior malignancy or recurrence of the tumor, ii) patients who underwent potentially curative surgery.

### Classification

All patients were classified according to Siewert’s classification and the AEGs were staged according to both the esophageal and gastric schemes of the seventh edition of the American Joint Committee on Cancer (AJCC) and UICC of malignant tumors using the histopathologic postoperative pTNM categories [5,10]. Therefore, AEGs with an epicenter within 5 cm of the EGJ and extension into the esophagus were classified and staged according to the esophageal scheme; AEGs which had an epicenter within 5 cm of the EGJ without extension into the esophagus were staged using the gastric carcinoma scheme.

### Statistical analysis

Statistical analyses were performed using SPSS 13.0. Consecutive data were presented as the mean ± standard deviation (SD). Categorical data were compared by a χ^2^ test or Fisher’s exact test. The means of the two groups were assessed with rank-sum test or *t*-test. Cumulative survival rates were generated by the Kaplan-Meier method. Survival curves and univariate significant factors were compared with the log-rank test. Cox proportional hazard models were constructed to investigate multivariable relationships of covariates with survival. All statistical tests were two-sided, and *P* values <0.05 were considered to be statistically significant.

## Results and discussion

### Patient and pathological characteristics

There were 330 males and 52 females in the 382 patients who had undergone curative radical resections (R0). Extended lymphadenectomy (two-field lymphadenectomy) was performed for type I adenocarcinomas and a systematic D2 lymphadenectomy was performed for type II and type III tumors. The ratio of male to female was 6.35:1 and the average age was 62.7 years old. Six of the 382 patients had type I (1.6%) adenocarcinomas, 220 had type II (57.6%), and 156 had type III (40.8%) adenocarcinomas.

The patient characteristics according to different Siewert types are presented in Table 1. There were no significant differences in age and gender among the subtypes. The tumor size of type III (64.7 mm) was significantly larger than that of type II (40.8 mm) and type I (35.0 mm). The esophageal invasion length was significantly different between the three types; it was longer for type I than for types II and III and the longest esophageal invasion was 50 mm in type I. Proximal gastrectomy with distal esophagectomy via laparotomy was more common in these patients than other approaches.

**Table 1:** Characteristics of patients with adenocarcinoma of the esophagogastric junction

**Siewert classification**	**Type I**	**Type II**	**Type III**	***P *value**
	**(n = 6)**	**(n = 220)**	**(n = 156)**	
Age (years)	65.5±9.7	62.4±8.6	63.0±8.4	>0.05
Male:Female	6:0	6.3:1	6.1:1	>0.05
Tumor size (mm)	35.0±7.7	40.8±16.8	64.7±27.8	<0.01
Esophageal invasion (mm)	28.3±16.3	10.7±9.1	6.0±7.2	<0.01
**Approaches**				<0.01
Transhiatal	2	169	136	
Transthoracic	2	42	17	
Transthoracoabdominal	2	9	2	
**Resection range**				<0.01
Total gastrectomy	0	6	15	
Proximal gastrectomy	6	214	141	
**TNM stage**				>0.05
Stage I	0	24	9	
Stage II	1	54	37	
Stage III	5	142	110	
**Histopathological grade**				>0.05
G1/2	3	115	65	
G3/4	3	105	91	
Neoadjuvant chemotherapy	0	11	3	>0.05
Adjuvant chemotherapy	1	40	16	>0.05
**Pattern of recurrence**				>0.05
Haematogenous	4	57	44	
Local recurrence	1	38	29	
Nodal recurrence	1	18	17	
Peritoneal recurrence	0	2	3	

The transthoracic technique was used the most for type I (66%) tumors, which included 50% of trans-left-thoracic and 50% of transthoracoabdominal approaches. In contrast, the transhiatal approach was common in type II and type III, the rates were 77% and 87%, respectively. For type I tumors, all six patients underwent proximal gastrectomy with distal esophagectomy (100%), whereas the rates of total gastrectomy in types II and III were 3% and 10%, respectively. According to the T category, N category, and AJCC stage, the distribution of the proportion in different Siewert types did not show a significant difference. According to the histopathological grade, the distribution of the proportion of well or moderately differentiated tumors was significantly higher in type II than in type III.

The average lymph node resection was 13.3±6.7 for type I, 13.5±7.1 for type II, and 15.1±7.4 for type III; 278 (72.8%) of the patients had lymph node metastasis. For those patients, the most common sites of nodal involvement were the paracardia (67.3%), lesser curvature (66.5%), greater curvature (12.9%), paraesophageal (2.9%), and left gastric artery (2.5%). In spite of the type I tumor, the frequency of lymph node metastasis was higher in patients with type II tumors than with type III tumors.

There was no significant difference in the rate of patients who received perioperative chemotherapy. The pattern of recurrence indicated that the most frequent type of recurrence was haematogenous and the most frequent site was the liver, followed by local recurrence and nodal recurrence; peritoneal recurrence was relatively infrequent. The pattern of recurrence in each subgroup did not show a significant difference. As for lymphovascular invasion, the rate was higher in T3-4 tumors (9.3%) than in T1-2 tumors (2.0%).

### Overall survival

**Figure 1:**
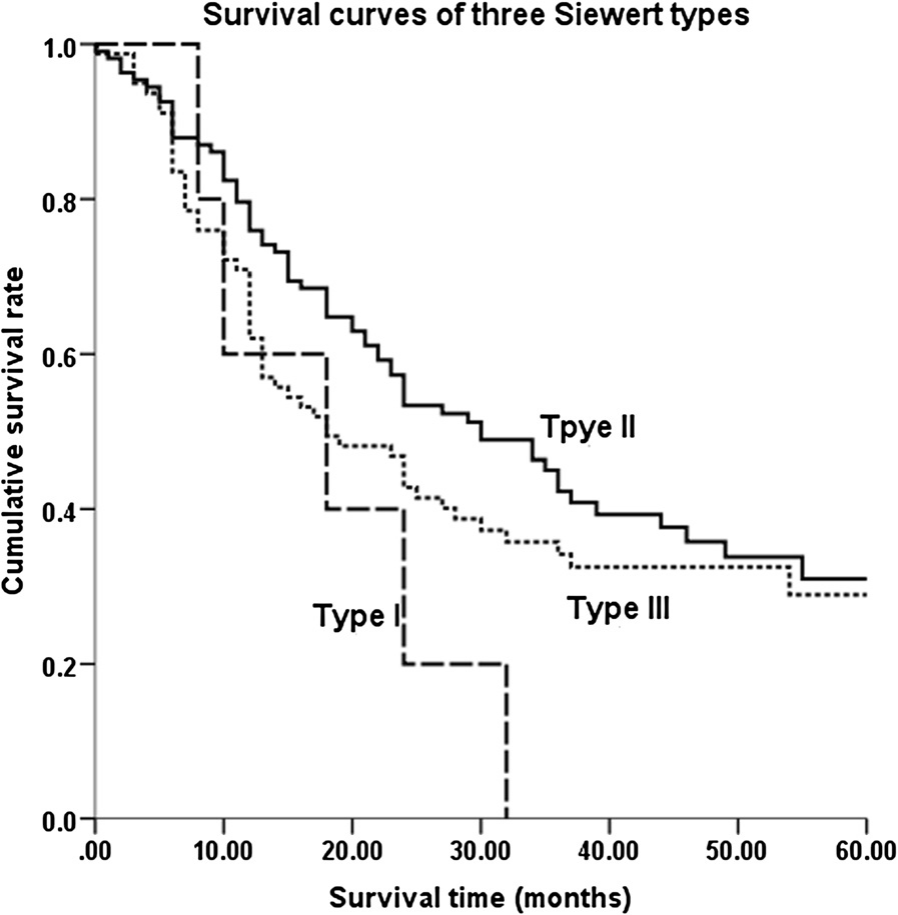
**Survival curves of Siewert types.** No significant difference in overall survival by subtypes was observed.

**Figure 2:**
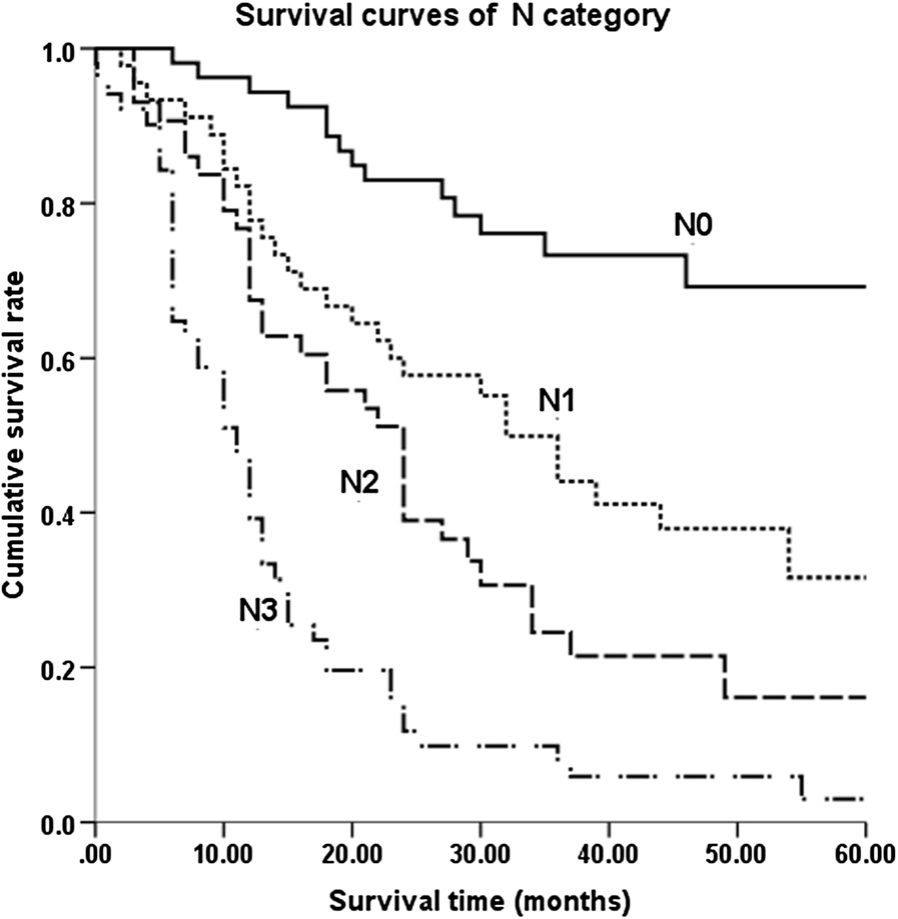
**Survival curves of lymph node metastasis.** There was a significant difference between N categories in patients.

**Figure 3:**
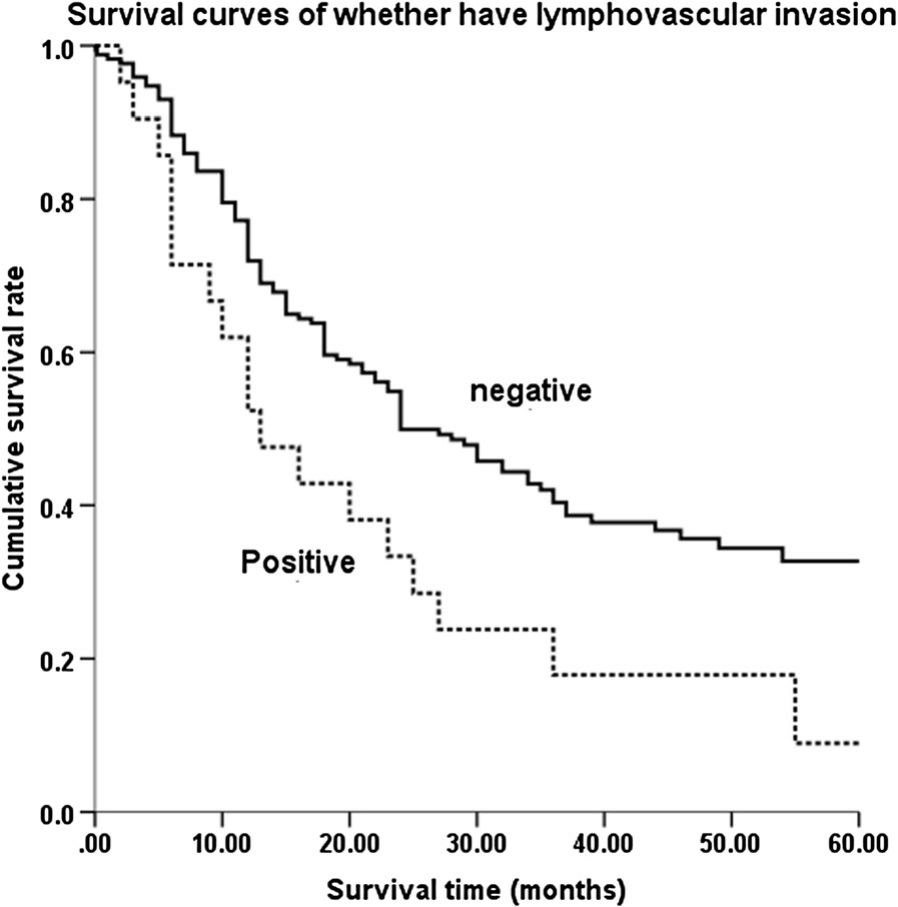
**Survival curves of lymphovascular invasion.** Patients whose lymphovascular invasion was negative had a significantly better 5-year survival rate than those were positive.

No significant difference was observed in the overall survival of different Siewert subtypes (*P* = 0.124; Figure 1). After pairwise comparison, type I versus type II was *P* = 0.480, type I versus type III was *P* = 0.288, and type II versus type III was *P =* 0.200. Kaplan-Meier survival analysis was used to assess ten other prognostic factors: age (<65 versus ≥65 years), gender, tumor maximal size (<50 mm versus ≥50 mm), esophageal invasion (positive versus negative), T stage, N stage, histopathological grade, lymphovascular invasion, operation methods, and combined organ resection. Univariate analysis revealed eight factors as having significant differences associated with the overall survival of AEG after surgery, namely age, tumor maximal size, total gastrectomy, combined organ resection, AJCC T stage, N stage, histopathological grade, and lymphovascular invasion. Multivariate analysis of these eight factors was performed to adjust the effects of covariates in a multivariate Cox proportional hazards model (Table 2). In this analysis, pathological lymph node stage, age, and the existence of lymphovascular invasion were the independent factors of prognosis of AEG following surgery. Therefore, it can be concluded that: i) patients who are older than 65 years old have a worse prognosis than those under 65 years old; ii) the greater the lymph node metastasis the lower overall survival rate (Figure 2); iii) patients with negative lymphovascular invasion have a better 5-year survival rate than those with positive lymphovascular invasion (Figure 3).

**Table 2:** Univariate and multivariate predictors of overall survival

	**Univariate analysis**		**Multivariate analysis**	
	***P *value**	**Hazard ratio (95% CI)**	***P *value**	**Hazard ratio (95% CI)**
Siewert classification				
Type I, II, III	>0.05	0.788–1.548		
Age (years)				
<65 or ≥65	<0.05	1.041–2.115	<0.05	1.051–2.206
Gender				
Male or Female	>0.05	0.628–1.707		
Tumor maximal size (mm)				
<50 or ≥50	<0.01	1.528–3.194	>0.05	0.878–1.920
Esophageal invasion				
Positive or Negative	>0.05	0.662–1.580		
T category				
T1-2 or T3-4	<0.01	2.085–7.655	>0.05	0.947–6.366
N category				
N0, N1, N2, N3	<0.01	1.702–2.401	<0.01	1.611–2.406
Histopathological grade				
G1/2 or G3/4	<0.01	1.367–2.416	>0.05	0.909–1.984
Combined organ resection				
With or Without	<0.01	1.285–3.707	>0.05	0.811–2.410
Lymphovascular invasion				
Positive or Negative	<0.05	1.117–3.042	<0.05	0.266–0.878
Resection range				
Total gastrectomy				
Proximal gastrectomy	<0.05	1.073–5.013	>0.05	0.512–2.530
Approaches				
Transthoracic				
Transhiatal				
Transthoracoabdominal	>0.05	0.740–1.498		

## Discussion

In this single-institution series of 382 AEG patients in China, the proportions of types I, II, and III carcinomas were 1.6%, 57.6%, and 40.8%, respectively. This showed a higher distribution of types II and III AEG in China compared with Western countries, but not with other Eastern nations [3,4,8,9]. Hosokawa et al. [9] found that, in Japan, 10 of 179 AEGs were type I patients; a retrospective analysis in Taiwan [8] did not reveal even one type I patient in 10 years. The lower frequency of type I AEG in Eastern countries may be explained by a lower prevalence of gastroesophageal reflux disease, a lower distribution of obese people, and a higher rate of *Helicobacter pylori* infection [11-13]. After R0 resection, the 5-year survival rates were 0% for type I, 29% for type II, and 23% for type III tumors. Although our data only included R0 resection, the outcomes seem worse than other prior reports. The 0% 5-year survival rate for type I patients was mainly due to most of the data being collected from stage III patients. The proportion of stage III AEG was 67.3%, which was higher than in other reports [8,14]; 83.3% of type I AEG was stage III. There was no significant difference in age and gender among the three types of AEG. However, according to the clinicopathological features, the three types were different; type III were more aggressive than type II, type III tumors were larger and deeper, with a higher rate of lymph node metastasis. This may indicate that type III carcinomas centered 2–5 cm below the EGJ might infiltrate the EGJ and are more difficult to detect early [9].

In the present study, the majority of patients with type II and III carcinomas underwent proximal gastrectomy with distal esophagectomy via an abdominal approach. In recent years, total gastrectomy has emerged as the standard procedure to treat types II and III AEG. However, in our hospital, most surgeons perform the operation ensuring a large enough non-infiltrating margin (5 cm), which might explain why proximal gastrectomy was performed in most patients. Only those who had large-sized tumors received a total gastrectomy in order to obtain a negative surgical margin. Thus, there is a connection between the large tumor size, more aggressive characteristics, and total gastrectomy, leading to a worse 5-year survival rate for total gastrectomy compared to proximal gastrectomy. Further, multivariate analysis (surgical procedure and tumor size) indicated that the surgical procedure is not the crucial factor (*P* = 0.106) while the survival benefits between different tumor size are significantly different (*P* <0.001). Thoracic surgeons were more likely to select the transthoracic approach to treat AEG; however, for type III patients, this might cause an insufficient lymphadenectomy. Therefore, an abdominal approach is more suitable for type III AEG.

The UICC sixth TNM classification did not include a criterion for AEG, which caused a confusion regarding the staging of AEG according to esophageal or gastric scheme criteria. Previous studies have usually chosen to treat type I as esophageal scheme, and type II and III as gastric scheme. The UICC seventh TNM classification has now defined the staging scheme criteria for AEG. Here we classified and staged the 382 AEG according to the latest criteria. It is noted that type I did not change staging scheme while some type II and III AEG changed from the gastric to the esophageal scheme; further, some tumors which extend into the esophagus are staged according to the esophageal scheme while they might originate from the gastric mucosa. AEGs might have different biological properties compared with genuine gastric and genuine esophageal cancers [15]. Therefore, further studies are required in order to ascertain if the latest scheme is suitable or not.

Multivariate analysis showed that lymph node metastasis, age, and the existence of lymphovascular invasion were independent prognostic indicators for AEG after R0 resection. Among the three factors, we demonstrated that lymph node metastasis was the strongest poor prognostic factor (odds ratio = 2.0). Our data showed that lymph nodes around the cardia, the lesser curvature of the proximal stomach, and along with the greater curvature of the proximal stomach had the highest rate of metastasis. This means that an appropriate resection of abdominal lymph nodes is very important to AEG patients [8], especially for the thoracic approach. In addition, although lymphovascular invasion was thought to precede or occur coincidently with lymph node metastasis [16], we found that in the advanced disease, lymphovascular invasion occurred at a considerably high rate. As a prognostic factor, the ratio of lymphovascular invasion increased with deeper carcinoma invasion and larger size causing a poor prognosis. Patients older than 65 years old had a poorer prognosis than younger patients. While most studies in the West indicated that lymph node metastasis and Siewert types were prognostic factors, they found type I patients had a significantly better overall survival rate than type II and III patients [17,18]. This might be partly due to a high distribution of types II and III AEG in Eastern countries compared with Western countries. The low incidence of type I AEG might cause some misleading or confusion in the overall survival rate.

## Conclusions

The distribution of the three types of tumor was found to be different from that reported in Western countries. Lymph node metastasis, lymphovascular invasion, and age were significant and independent factors of poor prognosis for AEG after R0 resection.

## Consent

Written informed consent was obtained from the patient for publication of this report and any accompanying images.

## Abbreviations

AEG: Adenocarcinomas of the esophagogastric junction; AJCC: American joint committee on cancer; EGJ: Esophagogastric junction; UICC: International union against cancer.

## Competing interests

The authors declare that they have no competing interests.

## Authors’ contributions

ZH and collected and evaluated the data. WW and CY re-analyzed the data. All authors participated in the conception and design of the study. All authors have read and approved the final manuscript.
